# Mixing Nulliparous and Multiparous Women in Randomised Controlled Trials of Preeclampsia Prevention Is Debatable: Evidence from a Systematic Review

**DOI:** 10.1371/journal.pone.0066677

**Published:** 2013-06-24

**Authors:** Emmanuel Simon, Agnès Caille, Franck Perrotin, Bruno Giraudeau

**Affiliations:** 1 INSERM, UMR-S 738, Paris, France; 2 CHRU de Tours, Service d’obstétrique, France, Université François Rabelais, Tours, France; 3 INSERM, CIC 202, Tours, France; Université François Rabelais, Tours, France; CHRU de Tours, Tours, France; Yale School of Public Health, United States of America

## Abstract

**Background:**

Nulliparity is a major risk factor of preeclampsia investigated in numerous trials of its prevention.

**Objective:**

We aimed to assess whether these trials considered nulliparity in subject selection or analysis of results.

**Search Strategy:**

01 April 2013 search of MEDLINE via PubMed, EMBASE and the Cochrane Library. 01 April 2013 search of trials registered in Clinicaltrials.gov.

**Selection Criteria:**

Randomised controlled trials and metaanalyses of preeclampsia prevention with no restriction to period of publication or language. Metaanalyses were selected to fully identify relevant trials.

**Data Collection and Analysis:**

One reader appraised each selected article/registered protocol using a pretested, standardized data abstraction form developed in a pilot test. For each article, he recorded whether both nulliparous and multiparous were included and, in case of mixed populations, whether randomisation was stratified, and whether subgroup analyses had been reported. For registered protocols, he only assessed whether it was planned to include mixed populations.

**Main Results:**

88 randomised controlled trials were identified, representing 83,396 included women. In 58 of the 88 articles identified (65.9%), preeclampsia was the primary outcome. In 31 of these (53.4%), the investigation combined nulliparous and multiparous women; only two reports in 31 (6.5%) stated that randomisation was stratified on parity and only four (12.9%) described a subgroup analysis by parity. Of the 30 registered trials, 20 (66.6%) planned to include both nulliparous and multiparous women.

**Conclusion:**

Parity is largely ignored in randomised controlled trials of preeclampsia prevention, which raises difficulties in interpreting the results.

## Introduction

Preeclampsia is defined as the association of pregnancy-induced hypertension and proteinuria of ≥300 mg/24 h after 20 weeks’ gestation [1] [2]. Preeclampsia is a common disease complicating 3% of pregnancies (4.1% in the first pregnancy and 1.7% in later ones) [3] and causing significant maternal and perinatal morbidity. A systematic review [4] highlighted that 10% to 15% of maternal deaths that occur every year worldwide are related to hypertensive complications of pregnancy. Preeclampsia is also a major cause of iatrogenic preterm delivery [2] [5]. Many randomised controlled trials (RCTs) of prevention and several reviews have assessed different interventions such as antiplatelet agents (e.g., aspirin) [6], calcium [7], antioxidants [8] or even garlic [9] or marine oil and other prostaglandin precursors [10]. The Cochrane Library contains several reviews dealing with prevention of preeclampsia.

Risk factors of preeclampsia have been largely investigated. Duckitt *et al*., [11] in their systematic review, listed risk factors that included (in decreasing pooled relative-risk order) antiphospholipids antibodies, previous history of preeclampsia, pre-existing diabetes, multiple pregnancy, nulliparity, family history, increased blood pressure, increased body mass index, and maternal age. Nulliparity holds a special place for three reasons. First, with a relative risk of 2.91 [11] and a prevalence of 44.6% among pregnancies [12], the population attributable risk can be estimated at 46.0% [13]. Thus, almost half of the preeclampsia episodes may be due to nulliparity. In terms of public health, nulliparity is the most important risk factor of preeclampsia and raises issues of prevention [14]. Second, by definition, multiparous women have an obstetric history as compared with nulliparous women; thus, women with previous preeclampsia have a 14.7% risk of the condition in the second pregnancy [3]. Therefore, in terms of prevention, identifying women at risk of preeclampsia naturally differs between nulliparous and multiparous women [14]. Third, in terms of pathophysiology, serious arguments suggest that mechanisms of preeclampsia differ between nulliparous and multiparous women [2]. A systematic review of underlying pathogenetic mechanisms of preeclampsia highlighted differences in immunological responses, angiogenic factor profile or reactivity to insulin resistance between early pregnancies and later ones [15]. Such differences may explain baseline risk differences between nulliparous and multiparous women and may also lead to heterogeneous preventive treatment effect [16].

Because of the specific place of the parity risk factor in preeclampsia, RCTs of prevention should be restricted to nulliparous or multiparous populations or at least be stratified by parity, [16] with *ad hoc* subgroup analyses. We aimed to assess whether such trials conform to this direction by undertaking a systematic review of published reports of RCTs and descriptions of registered trials [17]. We also aimed to illustrate how mixing these two populations in RCTs may impact both the nominal power of the trial and the interpretation of the results.

## Methods

### Systematic Literature Search and Study Selection

On 01 April 2013, we performed an electronic search of MEDLINE via PubMed, EMBASE and the Cochrane Library to identify reports of RCTs and metaanalyses of preeclampsia prevention with no restriction to period of publication or language.

Reports of RCTs in MEDLINE were searched with use of the terms “preeclampsia OR preeclampsia” and “prevention”, with a limitation to RCTs. Reports in EMBASE were identified by a “disease search” with the terms “preeclampsia OR pre-eclampsia”, with “prevention” in disease subheadings and a limitation to RCTs (including sub-terms; search terms had to be a major focus in retrieved articles). Reports were assessed by one of us (ES), who screened the titles and abstracts to identify relevant studies. Articles were selected if they reported results of a superiority prevention RCT with preeclampsia as an endpoint (either primary or secondary). We re-ran the electronic searches in MEDLINE and EMBASE databases with a limitation to metaanalyses.

We identified reports in the Cochrane Library by an advanced search using the terms “preeclampsia OR pre-eclampsia” and “prevention” in titles, abstracts or keywords. Reports were assessed by one of us (ES), who screened the titles and abstracts to determine whether the reports described results of metaanalyses of RCTs of preeclampsia prevention.

From reference lists of selected reports, we identified other randomised trials and associated reports. Reports were screened for duplicate publication (i.e., the same study described in several reports), and only the more detailed report was selected.

### Data Collection

We generated a standardized data collection form that was tested by the 2 reviewers (ES, AC) on a set of 10 randomly selected reports. The remaining reports were assessed by one of us (ES) in random order. Corresponding authors were not contacted. We collected the following data.

#### General characteristics of the study

We recorded the publication date, type of journal (general medical or obstetrical) and methodological characteristics as listed in the Delphi list [18].

#### Design and statistical issues

We collected data on the number of groups and whether and how a sample size calculation had been performed. We checked whether randomisation was reported as stratified (namely on parity), whether parity subgroup analyses had been performed, and if so, whether such analyses were pre-planned.

#### Studied population, intervention, outcomes

We recorded selection criteria known as risk factors [11], the nature of both the intervention and the control group, and whether pre-eclampsia was the primary endpoint.

### Search for Registered Trials and Data Extraction

We used the key words “preeclampsia” and “prevention” to search for RCT protocols that would assess preeclampsia prevention, with preeclampsia as a primary outcome, that were registered in Clinicaltrials.gov as of 01 April 2013. Moreover, we differentiated trials for which preeclampsia was the unique primary outcome and those for which preeclampsia was one adverse outcome of pregnancy among others. Then, for each trial we assessed whether the parity status was an eligibility criteria.

### Statistical Analysis

Descriptive data are presented as number and percentage for categorical variables and median (interquartile range) for continuous variables. Analyses involved use of R 2.9.1 (R Development Core Team. R: A language and environment for statistical computing. R).

### Ethical Issues

Institutional Review Board approval was not required for this systematic review.

### Mixing Nulliparous and Multiparous Women: Impact on Power and Results Interpretation

We considered a hypothetical RCT mixing primiparous and multiparous women, with an *a priori*-specified primiparous proportion. A sample size calculation was performed for a nominal power of 90% (or 80%). With this sample size, we derived the power of the trial, if the primiparous proportion was different from that *a priori* specified.

Considering a preventive effect common to both nulliparous and multiparous women, we calculated the associated numbers needed to treat (NNT). We also calculated the NNT for a trial in which nulliparous and multiparous women would have been mixed. We then varied the proportion of nulliparous women to illustrate its impact on the NNT.

## Results

### Published Reports

#### Selection results

**Figure 1:**
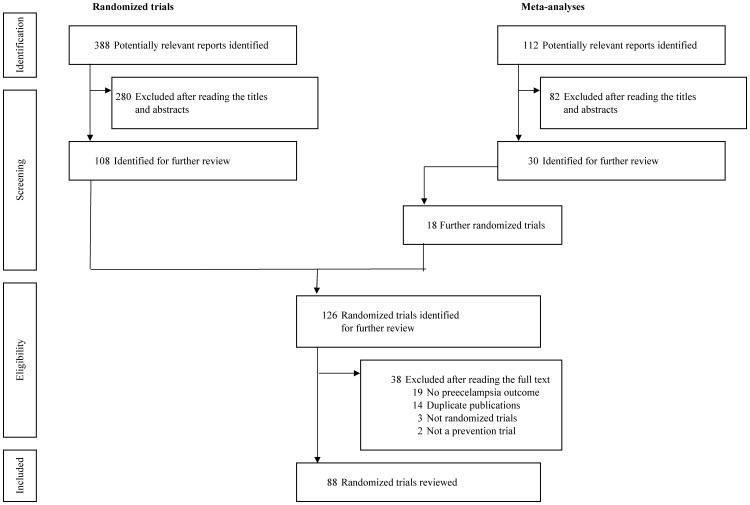
Selection of articles.

The search strategies for RCTs and metaanalyses generated 388 and 112 reports, respectively. Of them, 88 were eligible and evaluated (Figure 1). No translation was required.

#### Trial characteristics (Table 1)

**Table 1:** General characteristics of reports of randomised controlled trials of preeclampsia prevention.

	Total reports (n = 88)	Trials of nulliparous women (n = 34)		Trials of multiparous women (n = 7)		Trials of both mulliparous and multiparous women (n = 47)	
**Journal type**							
General medical	27 (30.7)	11 (32.4)		1 (14.3)		15 (31.9)	
Specialty	61 (69.3)	23 (67.6)		6 (85.7)		32 (68.1)	
**Publication date**							
1985–1989	5 (5.7)	2 (5.9)		0		3 (6.4)	
1990–1994	15 (17.0)	8 (23.5)		1 (14.3)		6 (12.8)	
1995–1999	24 (27.3)	11 (32.4)		1 (14.3)		12 (25.5)	
2000–2004	11 (12.5)	4 (11.8)		0		7 (14.9)	
2005–2009	16 (18.2)	6 (17.6)		2 (28.6)		8 (17.0)	
2010	17 (19.3)	3 (8.8)		3 (42.9)		11 (23.4)	
**Delphi List**							
*Was a method of randomisation performed?*							
Yes	88 (100.0)	34 (100.0)		7 (100.0)		47 (100.0)	
No	0	0		0		0	
Don’t know	0	0		0		0	
*Was the treatment allocation concealed?*							
Yes	43 (48.9)	10 (29.4)		4 (57.1)		29 (61.7)	
No	4 (4.5)	2 (5.9)		0		2 (4.3)	
Don’t know	41 (46.6)	22 (64.7)		3 (42.9)		16 (34.0)	
*Were the groups similar at baseline regarding the most important prognostic indicators?*							
Yes	79 (89.8)	31 (91.2)		7 (100.0)		41 (87.2)	
No	1 (1.1)	0		0		1 (2.1)	
Don’t know	8 (9.1)	3 (8.8)		0		5 (10.6)	
*Were the eligibility criteria specified?*							
Yes	85 (96.6)	33 (97.1)		7 (100.0)		45 (95.7)	
No	3 (3.4)	1 (2.9)		0		2 (4.3)	
Don’t know	0	0		0		0	
*Was the outcome assessor blinded?*							
Yes	61 (69.3)	28 (82.4)	2 (28.6)		31 (66.0)		
No	23 (26.1)	5 (14.7)	4 (57.1)		14 (29.8)		
Don’t know	4 (4.5)	1 (2.9)	1 (14.3)		2 (4.3)		
*Was the care provider blinded?*							
Yes	58 (65.9)	28 (82.4)	1 (14.3)		29 (61.7)		
No	26 (29.5)	5 (14.7)	5 (71.4)		16 (34.0)		
Don’t know	4 (4.5)	1 (2.9)	1 (14.3)		2 (4.3)		
*Was the patient blinded?*							
Yes	64 (72.7)	30 (88.2)	2 (28.6)		32 (68.1)		
No	23 (26.1)	3 (8.8)	5 (71.4)		15 (31.9)		
Don’t know	1 (1.1)	1 (2.9)	0		0		
*Were point estimates and measures of variability presented for the primary outcome measures?*							
Yes	86 (97.7)	33 (97.1)	7 (100.0)		46 (97.9)		
No	0	0	0		0		
Don’t know	2 (2.3)	1 (2.9)	0		1 (2.1)		
*Did the analysis include an intention-to-treat analysis?*							
Yes	49 (55.7)	20 (58.8)	6 (85.7)		23 (48.9)		
No	36 (40.9)	13 (38.2)	1 (14.3)		22 (46.8)		
Don’t know	2 (2.3)	1 (2.9)	0		1 (2.1)		
**Design**							
2 groups	83 (94.3)	32 (94.1)	6 (85.7)		45 (95.7)		
>2 groups	5 (5.7)	2 (5.9)	1 (14.3)		2 (4.3)		
**Sample size calculation reported**	61 (69.3)	25 (73.5)	5 (71.4)		31 (66.0)		
**Sample size, median (IQR)**	184 (98–689)	224(101–1027)	135 (98–150)		208 (77–751)		

Data are number (percentages), except for sample size. IQR,interquartile rang.

About one-third of the trials (27 [30.7%]) were published in general medical journals. Methodological characteristics were generally poorly reported and were issues related to allocation concealment, blinding, or the intention-to-treat principle.

#### Design and statistical issues (Table 1)

Most trials, 83 (94.3%), were parallel-group randomised trials with two groups. About one-third of the articles did not report a sample size calculation. The median sample size (interquartile range) was 184 (98–689).

#### Studied population, intervention, outcomes (Table 2)

**Table 2:** Characteristics of reports of randomised controlled trials specific to preeclampsia prevention.

*(a) All articles*				
	Total reports (n = 88)	Trials of nulliparous women (n = 34)	Trials of multiparous women (n = 7)	Trials of both mulliparous and multiparous women (n = 47)
**Selection criteria - Risk factors at booking, as listed in Duckitt ** ***et al***				
Antiphospholipid antibodies	3 (3.4)	1 (2.9)	0	2 (4.3)
Previous preeclampsia	28 (31.8)	-	5 (71.4)	23 (48.9)
Pre-existing diabetes	8 (9.1)	0	0	8 (17.0)
Twin pregnancy	7 (8.0)	0	0	7 (14.9)
Nulliparity ^(a)^				5 (10.6)
Family history of preeclampsia	7 (8.0)	1 (2.9)	0	6 (12.8)
Body mass index	6 (6.8)	0	0	6 (12.8)
Systolic blood pressure	4 (4.5)	0	0	4 (8.5)
Maternal age ≥40	4 (4.5)	0	0	4 (8.5)
Pre-existing hypertension	20 (22.7)	1 (2.9)	0	19 (40.4)
Renal disease	7 (8.0)	1 (2.9)	0	6 (12.8)
Chronic autoimmune disease	0	0	0	0
Time between pregnancies	0	0	0	0
**Experimental group (non-exclusive)**				
Aspirin	35 (39.8)	13 (38.2)	1 (14.3)	21 (44.7)
Calcium	15 (17.0)	13 (38.2)	0	2 (4.3)
Antioxidants	16 (18.2)	5 (14.7)	0	11 (23.4)
Other	26 (29.5)	4 (11.8)	7 (100.0)	15 (31.9)
**Control group**				
Placebo	62 (70.5)	31 (91.2)	2 (28.6)	29 (61.7)
Usual care	21 (23.9)	3 (8.8)	4 (57.1)	14 (29.8)
Other	5 (5.7)	0	1 (14.3)	4 (8.5)
**Primary outcome**				
Clearly reported	73 (83.0)	28 (82.4)	6 (85.7)	39 (83.0)
If yes, defined as preeclampsia	58 (65.9)	24 (70.6)	3 (42.9)	31 (66.0)
Data are number (percentages) – (a) Nulliparity considered as a risk factor in trials mixing nulliparae and multiparae				
***(b) Articles with preeclampsia reported as the primary outcome***				
	**Total reports (n = 58)**	**Trials of nulliparous women (n = 24)**	**Trials of multiparous women (n = 3)**	**Trials of both mulliparous and multiparous women (n = 31)**
**Selection criteria - Risk factors at booking, as listed in Duckitt ** ***et al***				
Antiphospholipid antibodies	4 (6.9)	1 (4.2)	0	3 (9.7)
Previous preeclampsia	19 (32.8)	0	2 (66.7)	17 (54.8)
Pre-existing diabetes	8 (13.8)	0	0	8 (25.8)
Twin pregnancy	5 (8.6)	0	0	5 (16.1)
Nulliparity ^(a)^				3 (9.7)
Family history of preeclampsia	3 (5.2)	0	0	3 (9.7)
Body mass index	1 (1.7)	0	0	1 (3.2)
Systolic blood pressure	7 (12.1)	0	1 (33.3)	6 (19.4)
Maternal age ≥40	6 (10.3)	0	1 (33.3)	5 (16.1)
Pre-existing hypertension	15 (25.9)	0	0	14 (45.2)
Renal disease	6 (10.3)	1 (4.2)	0	5 (16.1)
Chronic autoimmune disease	4 (6.9)	1 (4.2)	1 (33.3)	3 (9.7)
Time between pregnancies	0	0	0	0
**Experimental group (non-exclusive)**				
Aspirin	25 (43.1)	10 (41.7)	0	15 (48.4)
Calcium	14 (24.1)	9 (37.5)	1 (33.3)	4 (12.9)
Antioxidants	9 (15.5)	3 (12.5)	0	6 (19.4)
Other	11 (19.0)	3 (12.5)	2 (66.6)	6 (19.4)
**Control group**				
Placebo	47 (81.0)	22 (91.7)	2 (66.6)	23 (74.2)
Usual care	9 (15.5)	2 (8.3)	1 (33.3)	6 (19.4)
Other	2 (3.4)	0	0	2 (6.5)

Data are number (percentages) – (a) Nulliparity considered as a risk factor in trials mixing nulliparae and multiparae.

The risk factor most considered an inclusion criterion in trials was previous preeclampsia (31.8%), then pre-existing hypertension (22.7%). Nulliparity was stated as a risk factor in 10.6% of the reports for trials mixing nulliparous and multiparous women. The studied interventions were mostly aspirin (35 trials, 39.8%), calcium (15 trials, 17.0%) and antioxidants (16 trials, 18.2%). The primary outcome was clearly reported in 73 articles (83.0%) and was preeclampsia in 58 (79.5%) of these reports.

#### Parity as a selection criterion, stratification variable and subgroup

**Figure 2:**
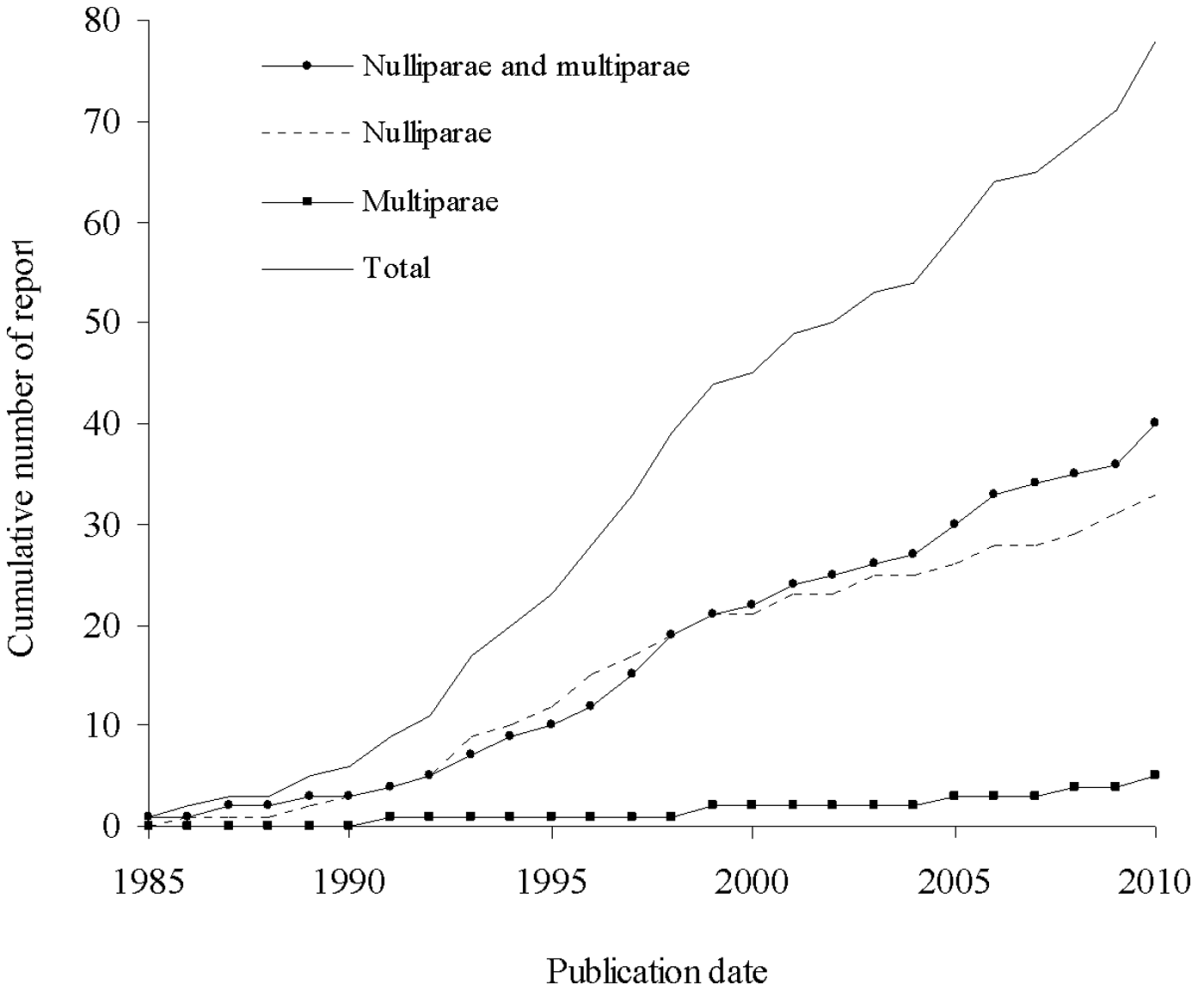
Number of reports of randomised trials of preeclampsia prevention by publication date.

More than half of the trials (47 [53.4%]) included both nulliparous and multiparous women, 34 (38.6%) only nulliparous women, and the last seven (8.0%) only multiparous women. For the trials with preeclampsia as the primary outcome (58 trials), 31 (53.4%) included both nulliparous and multiparous women. Figure 2 displays the number of RCTs by date of report publication: trials including both nulliparous and multiparous women were still being planned recently. A sample size calculation was reported in 61 (69.3%) of the articles, and in 45 (73.8%) of these, preeclampsia was the primary outcome. Among the 31 trials mixing nulliparous and multiparous women with preeclampsia as the primary outcome, randomisation was described as stratified on parity in only two (6.5%) reports; the proportion of nulliparous women was specified in 24 of these reports (77.4%). In these 24 trials, the median proportion of nulliparous women was 31.5% (interquartile range 23.1–49.6%), thus illustrating a highly variable proportion among trials. Finally, only four reports (12.9%) described parity subgroup analyses. Only one report described these subgroups analyses as planned before the trial start, which was one of the two trials reporting stratified randomisation by parity.

### Registered Trials

The search for registered trials identified 72 protocols, with 30 eligible for inclusion: 14 with preeclampsia as the unique primary outcome and 16 with preeclampsia as one component of a composite outcome. Among these 30 trials, 20 (66.6%) planned to include mixed populations (i.e., nulliparous and multiparous women), eight of the 14 trials with the unique primary outcome and 12 of the 16 trials with a composite primary outcome.

### Mixing Nulliparous and Multiparous Women: Impact on Power and Results Interpretation

**Impact on power:** Let us assume an *a priori* expected proportion of 44.6% of nulliparous women [12] with baseline risk of 4.1% (1.7% for multiparous women [3]) and an *a priori*-postulated relative risk of 0.9 [19]. In nulliparous women were 20% instead of 44.6%, the trial power would decrease from a nominal value of 90% to 82% (or from a nominal value of 80% to 70%).

#### Impact on NNT interpretation

If we hypothesize a preventive effect of aspirin for preeclampsia of 0.9 (relative risk) [19]for both nulliparous and multiparous women, because of a baseline risk of 4.1% for the former and 1.7% for the latter [3], the NNT is actually 244 and 588, respectively. However, if nulliparous and multiparous women are mixed, with only a global result reported (i.e. no subgroup results), the NNT will be estimated at 361 if the proportion of nulliparous women is 44.6% and 459 if the proportion is 20%.

## Discussion

We reviewed 88 reports of RCTs assessing preventive treatments of preeclampsia. In half of the reports, parity was not a selection criterion, which led to mixing the nulliparous and multiparous populations. Such a practice is still in force, with 20 of 30 registered trials reporting this practice. Among published reports of RCTs that included nulliparous and multiparous women and reported preeclampsia as the primary outcome, only four (12.9%) reported parity subgroup analyses.

Including patients with different baseline risks in an RCT is common [20]. Nevertheless, averaging effects across subgroups defined according to baseline risk levels can be misleading for two reasons. First, if the treatment relative effect is common to all subgroups, the absolute treatment effect (expressed as a NNT) varies across subgroups [21] [22]. Second, treatment relative effect may vary across risk-level subgroups, with a greater treatment effect in high-risk subgroups or vice versa [23]. For RCTs of preeclampsia prevention, this point is all the more relevant, if we accept that mechanisms of preeclampsia differ between nulliparous and multiparous women [15]. Therefore, investigating nulliparous and multiparous women together and averaging prevention treatment effects across parity subgroups may be misleading. At least, if trialists mix these populations, randomisation should be stratified, statistical analysis should take into account this stratification [24] and a subgroup analysis should be correctly performed and reported [25].

Otherwise, mixing nulliparous and multiparous women in an RCT of preeclampsia prevention raises issues for translating results into clinical practice [26]. Thus, identifying multiparous women at high-risk of preeclampsia (e.g., with a history of preeclampsia) and treatment is easy; treating preeclampsia in a pregnant woman just because she is nulliparous is probably difficult and cost-ineffective (because it concerns millions of women). In practical terms, nulliparity cannot be viewed as one preeclampsia risk factor among others, which also suggests the mandatory identification of biomarkers and Doppler variables that can reliably predict the onset of preeclampsia in nulliparous women, as well as the definition of a predictive model, as was recently done by North *et al* [14].

Our study has limitations. First, only one reader assessed papers. Nevertheless, regarding the elements extracted from the published papers, most of them were objective elements (e.g., whether included populations mixed nulliparous and multiparous women, whether randomization was stratified, whether a subgroup analysis was reported …). Otherwise, one major limitation is discrepancies between real and reported methods. Some deficiencies may simply appear because of poor reporting, which does not necessarily mean that the methods were not used [27] [28]. Therefore, we may have underestimated the number of RCTs with parity stratification (among trials including both nulliparous and multiparous women). In the same way, parity subgroup analyses may have been done but not reported. Concerning registered trials, we could not assess whether stratified randomisation or subgroup analyses was planned because of lack of information.

In conclusion, in RCTs of preeclampsia prevention, nulliparity is often considered as one risk factor among others. This practice is questionable and raises issues when interpreting trial results and translating them into clinical practice. Mixing different populations in a trial is a debatable issue, and the results we observed in the field of preeclampsia prevention may also occur in other fields.
